# Arbuscular mycorrhizal fungi improve the competitive advantage of a native plant relative to a congeneric invasive plant in growth and nutrition

**DOI:** 10.1002/ece3.11459

**Published:** 2024-05-21

**Authors:** Kaiping Shen, Yuejun He, Tingting Xia, Yun Guo, Bangli Wu, Xu Han, Hongchun Chen, Yan Zhao, Pan Wu, Yuan Liu

**Affiliations:** ^1^ Forestry College, Research Center of Forest Ecology Guizhou University Guiyang China; ^2^ College of Eco‐Environmental Engineering Guizhou Minzu University Guiyang China; ^3^ College of Resources and Environmental Engineering Guizhou University Guiyang China; ^4^ Guizhou Provincial Water Conservancy Research Institute Guiyang China; ^5^ College of Life Science Guizhou University Guiyang China

**Keywords:** arbuscular mycorrhizal fungi, competition, nutrition acquisition, plant growth, plant invasion

## Abstract

Plant invasions severely threaten natural ecosystems, and invasive plants often outcompete native plants across various ecosystems. Arbuscular mycorrhizal (AM) fungi, serving as beneficial microorganisms for host plants, can greatly influence the competitive outcomes of invasive plants against native plants. However, it remains unclear how AM fungi alter the competitive balance between native and invasive species. A competitive experiment was conducted using an invasive *Eupatorium adenophorum* paired with a native congener *Eupatorium lindleyanum*. Specifically, both species were inoculated with (M^+^) or without (M^−^) the fungus *Glomus etunicatum* under intraspecific (Intra‐) and interspecific (Inter‐) competition. Plant traits were measured and analyzed regarding the growth and nutrition of both species. The results exhibited that the AM fungus significantly increased the height, diameter, biomass, C, N, and P acquisition of both the invasive *E. adenophorum* and the native *E. lindleyanum*. The root mycorrhizal colonization and the mycorrhizal dependency of native *E. lindleyanum* were greater than those of invasive *E. adenophorum*. Under M^+^, the Inter‐competition inhibited the growth and nutrition of invasive *E. adenophorum* compared to the Intra‐ competition. Further, native *E. lindleyanum* exhibited higher competitiveness than invasive *E. adenophorum* in growth and nutrition. Meanwhile, the AM fungus significantly improved the competitiveness of native *E. lindleyanum* over invasive *E. adenophorum*. In conclusion, AM fungus improved the competitive advantage of native *E. lindleyanum* over invasive *E. adenophorum* in growth and nutrition, potentially contributing to native species competitively resisting the invasion of exotic species. These findings emphasize the importance of AM fungi in helping native plants resist the invasion of exotic plants and further contribute to understanding plant invasion prevention mechanisms.

## Graphical abstract

Mutualistic interactions with AM fungi greatly affect the outcome of invasive plants competing against native plants. We examined the effects of AM fungi on the competition between invasive plants and congeneric native plants under intra‐ and interspecific competition. The results indicate that AM fungi significantly improved the competitive advantage of native plants over congeneric exotic plants in growth and nutrition, which contributed to native plants competitively resisting exotic plants.

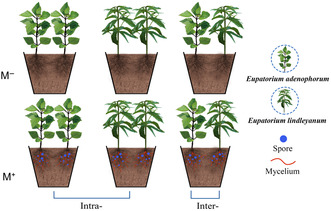

## 1 INTRODUCTION

Biological invasion inevitably causes great damage to invaded sites (Diagne et al., 2021; Wang et al., 2022). In particular, plant invasions often result in a substantial reduction in biodiversity and stability, posing severe threats to native plant communities (Hansen et al., 2021; Livingstone et al., 2020; Zubek et al., 2022). Furthermore, they can alter soil physicochemical properties through plant–soil microbial interactions (Stefanowicz et al., 2019). Therefore, the prevention of exotic plant invasions is widely regarded as a priority biocontrol measure across several regions worldwide. Darwin (1859) proposed the “Darwinian naturalization hypothesis,” suggesting that invaders with greater phylogenetic distance from natives are more likely to naturalize due to limited niche overlap, resulting in weak interspecific competition (Daehler, 2001). Similarly, MacArthur and Levins (1967) raised the “limiting similarity hypothesis,” suggesting that invasive plants with high similarity to native plants are less likely to successfully establish due to similar resource acquisition strategies. Nevertheless, Li et al. (2015) discovered that invasive plants closely related to native species were more likely to establish themselves in resident communities after investigating invasion dynamics in 480 plots over 40 years. Consequently, how native plants resist invasion by congeneric exotic plants remains pending. In fact, invasive plants inevitably generate competitive interactions with native plants when they invade a new habitat, whereas the native plants have the potential to resist the intrusion when their habitat is threatened (Maron & Marler, 2007; Wang et al., 2023). Therefore, it is necessary to explore how native species resist the invasion by exotic plants to understand the mechanisms underlying successful invasion by exotic plants.

The competitive advantage between invasive and native plants is considered a key factor in determining whether an invasive plant can successfully invade or whether native plants can successfully resist the invasion of invasive plants (Beaury et al., 2020; Qiu et al., 2023; Vila & Weiner, 2004). Research suggests that the competitive advantage of both invasive and native plants, for mycorrhizal species, largely depends on their associations with symbiotic microorganisms (Aslani et al., 2019; Koch et al., 2011). Arbuscular mycorrhizal (AM) fungi, a group of soil microorganisms, form symbiotic partnerships with over two‐thirds of terrestrial plants, providing benefits to the host plant, such as water and nutrient transfer in exchange for photosynthate (Smith & Read, 2008). AM fungi can influence the competitive dynamics of invasive and native plants by influencing the uptake and transport of key resources such as carbon (C), nitrogen (N), and phosphorus (P) (Daisog et al., 2012; Merrild et al., 2013). For example, Řezáčová et al. (2020) concluded that AM fungi disproportionately allocated mycorrhizal benefits, such as nutrient supply, between invasive *Echinops sphaerocephalus* and native *Inula conyzae*, thus regulating the competitive advantage of invaders and natives. In addition, Shen et al. (2020) and Xia et al. (2020) observed that AM fungi conferred greater growth and nutrient competition for the invasive *Ageratina adenophora* compared to the native *Artemisia annua* through mycorrhizal networks. Accordingly, AM fungi can partially explain the successful invasion of plant invaders (Bunn et al., 2015). However, the roles of AM fungi on invasive plants can range from positive to negative, which are affected by plant and fungal species, soil physicochemical properties, mycorrhizal associations, and other factors (Aslani et al., 2019; Sun et al., 2022; Waller et al., 2016). Therefore, the role of AM fungi in altering the competitive balance between invasive and native plants remains unclear.

In general, intraspecific competition is expected to be more intense than interspecific competition for any pair of species, as conspecific individuals have more similar resource requirements than heterospecific individuals (Adler et al., 2018; Bengtsson et al., 1994; Hart & Marshall, 2009). However, diametrically opposite trends can occur for invasive and native plants, where interspecific competition may actually be more intense than intraspecific competition (Sheppard & Burns, 2014). Research has demonstrated that fierce competition limits the ability of plants to access resources (Yang et al., 2019). Therefore, the intensity of intra‐ and interspecific competition can impact the competitive balance between invasive and native plants by mediating differences in growth between the two species. In particular, AM fungi can greatly affect intra‐ and interspecific interactions in both invasive and native plants, thereby regulating plant competition intensity (Cheng, Cao, et al., 2022; Weremijewicz et al., 2018). For instance, Zhang et al. (2023) speculated that AM fungi alleviated the interspecific competition intensity between *Leymus chinensis* and invasive weed *Stellera chamaejasme*, promoting the underground growth of *L. chinensis*. Sun et al. (2022) revealed that invasive Asteraceae plants exhibit higher interspecific competition intensities compared to natives, and AM fungi significantly altered the intra‐ and interspecific competition intensities of invasive and native plants. Therefore, it is reasonable to assume that the different roles of AM fungi in intra‐ and interspecific competition for invasive and native plants can influence the competitive balance between the two species (Cheng et al., 2019; Moora & Zobel, 1996).

*Eupatorium adenophorum* is widely recognized as one of the most destructive invasive plant species worldwide, posing a severe threat to the economic and ecological balance of many regions worldwide (He et al., 2015; Zhang et al., 2018), including southwest China. Specifically, invasive *E. adenophorum* causes severe damage to local ecosystems, resulting in a reduction in biodiversity (Fang et al., 2021). Furthermore, livestock, especially horses, also suffer negative impacts from invasive *E. adenophorum*, resulting in agricultural and forestry economic losses (Shapter et al., 2023). Previous research has indicated that AM fungi play a positive role in the invasion of *E. adenophorum* (Shen et al., 2020; Xia et al., 2020). However, the specific mechanism of this invasion remains unclear, especially lacking a comparison with native congeneric species. *Eupatorium lindleyanum* is a native congener of invasive *E. adenophorum*, and both species exhibit a beneficial symbiotic association with AM fungi (Xiao et al., 2014; Zheng et al., 2021). Therefore, we conducted a competition experiment, pairing the invasive species *E. adenophorum* with its native congeneric species *E. lindleyanum*, to investigate the role of AM fungi in altering the competitive balance between invasive and native plants and further understand the regulatory mechanisms of AM fungi in plant invasion. Based on previous studies that highlight the competitive advantage of invasive plants over native plants and the promotion of invasion by AM fungi (Dong et al., 2021; Shen et al., 2020; Zhang et al., 2018, 2022), we hypothesized the following: (1) invasive plants exhibit higher competitiveness than native plants, with AM fungi promoting the competitive advantage of the invasive species (H1). Additionally, one of the initial interactions invasive plants experience upon introduction is competition with the recipient community for limited resources, potentially leading to more intense interspecific competition between the invasive plant and native plants compared to intraspecific competition (Sheppard & Burns, 2014). Thus, we hypothesized that (2) the intensity of interspecific competition between invasive and native plants is greater than the intraspecific competition within invasive or native plant populations (H2).

## 2 MATERIALS AND METHODS

### 2.1 Experimental design

**FIGURE 1:**
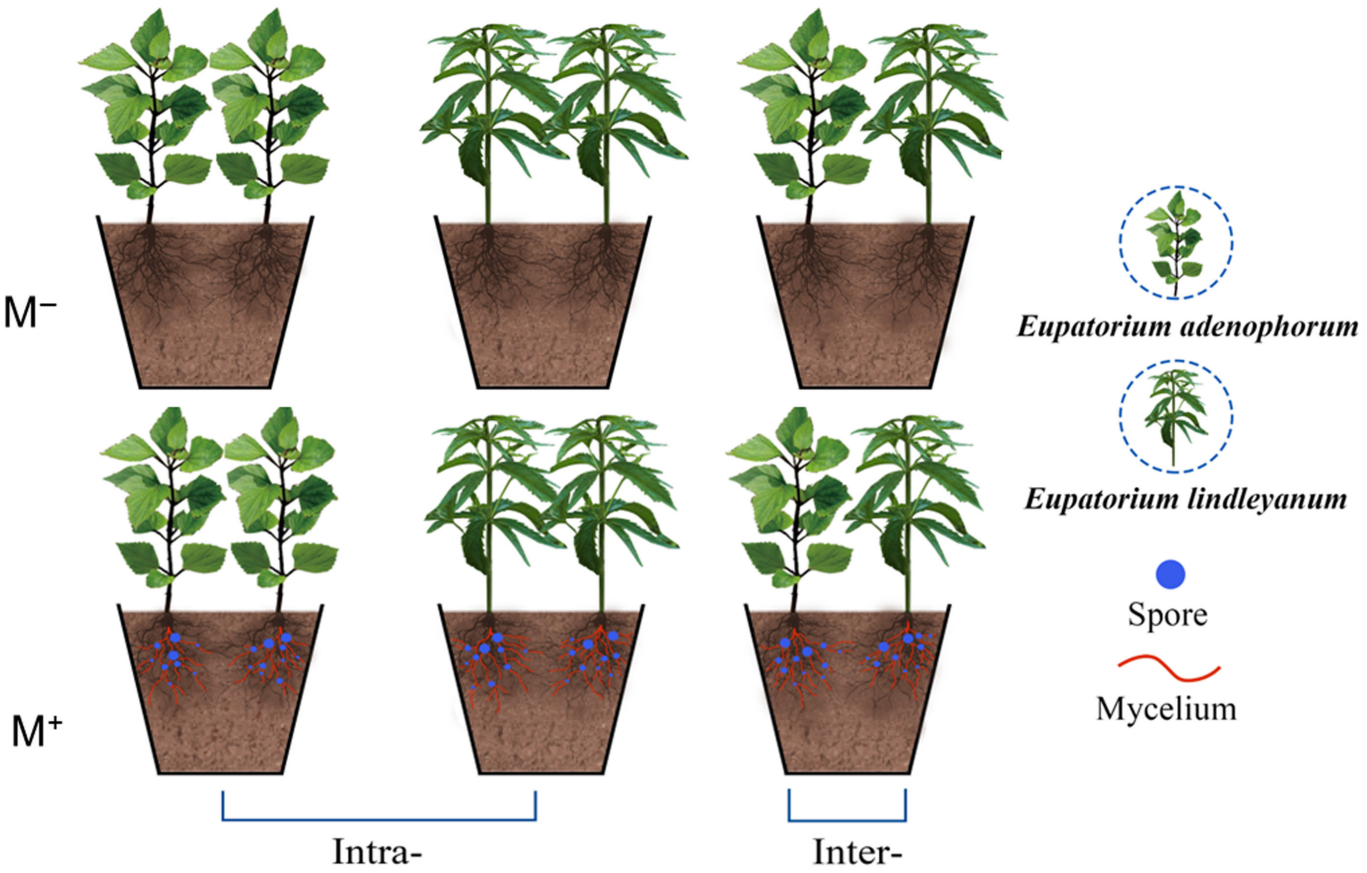
Schematic diagram of the experimental design. Two plant species (invasive plant *Eupatorium adenophorum* and native plant *Eupatorium lindleyanum*) were grown in intraspecific (Intra‐) competition or interspecific (Inter‐) competition, and with (M^+^) or without (M^−^) inoculation of AM fungus *Glomus etunicatum* in autoclaved soil.

An experiment (Figure 1) was established using plastic flowerpots (22 cm × 20 cm × 28 cm, caliber × bottom diameter × height). Each plastic flowerpot had a round hole with a diameter of 1 cm at the bottom to prevent water pooling. The experiment was a crossed design of two treatments involving arbuscular mycorrhizal (AM) fungus and competition. The AM fungus treatments included inoculation (M^+^) or non‐inoculation (M^−^) with *Glomus etunicatum*. The competition treatments encompassed intraspecific (Intra‐) competition and interspecific (Inter‐) competition. Specifically, the Intra‐ competition was achieved by planting two conspecific individuals of *E. adenophorum* or *E. lindleyanum* in a single pot, and the Inter‐ competition was achieved by planting one seedling of *E. adenophorum* alongside one seedling of *E. lindleyanum* in a pot. Incorporating two plants in each plot helps exclude the density dependence.

The seeds of exotic invasive *E. adenophorum* and native *E. lindleyanum* were picked in Anshun City, Guizhou Province, China. Store the seeds at room temperature until use. The *G. etunicatum* inoculum was bought from the Institute of Nutrition Resources, Beijing Academy of Agricultural and Forestry Sciences, No. BGA0046, and then it was propagated for the experiment. Specifically, the inoculum was grown for 4 months in sterilized soil substrate using *Trifolium repens*. After removing the *T. repens*, naturally air‐dried soil was collected and kept at 4°C until it was needed. In addition, the soil substrate was collected from Huaxi District, Guiyang City, Guizhou Province, China. After the removal of residual litter and roots, the soil was naturally air‐dried and then autoclaved at 0.14 Mpa at 121°C for 1 h. The soil was measured before the start of the experiment by adopting the methods described by Bao (2000) and contained 7.49 pH, 37.14 g/kg soil organic carbon (SOC), 0.503 g/kg total nitrogen (TN), 410.576 mg/kg available nitrogen (AN), 0.704 g/kg total phosphorus (TP), 5.023 mg/kg available phosphorus (AP), 6.489 g/kg total potassium (TK), and 195.443 mg/kg available potassium (AK).

Further, the seeds of exotic *E. adenophorum* and native *E. lindleyanum* were sown into the pot containing 2 kg of soil substrate. Subsequently, 50 g of inoculum, consisting of soil, hyphal pieces, and colonized root segments with about 100 spores per 10 g, was added to the M^+^ treatment and 50 g of autoclaved inoculum was added to the M^−^ treatment. To maintain uniform microflora in M^+^ and M^−^ treatments, 10 mL of filtrate filtered by 20 μm membrane from 50 g unsterilized inoculum was added to the M^−^ treatment. The experiment contained two AM fungus treatments, three competition treatments involving two plant species, and six replicates, totaling 36 pots. Initially, 3–5 seeds were sown in each flowerpot. After germination, two seedlings of equal size were left in each pot (approximately 1 month after sowing the seeds). Subsequently, the retained plants were allowed to grow for an additional approximately 2 months in a plastic greenhouse with an open underside to permit air circulation at Guizhou University (106°220 E, 29°490 N, 1120 m above sea level), with water once a day until the day before harvest. All plants were cultivated for a total of approximately 3 months. Finally, we harvested and measured all plant and soil materials.

### 2.2 Measurements of growth and nutrition indicators of harvested plant and soil

Root mycorrhizal colonization was determined according to the method described by Giovannetti and Mosse (1980). The height was measured with a 100 cm ruler, the diameter was measured with a standard vernier caliper, and the total biomass was measured by weighing individual plants, including root, stem, and leaf, after drying them at 75°C for 48 h. Additionally, soil pH was measured by the potentiometric method; SOC of soil and plant tissue (including root, stem, and leaf) C concentrations of dried plant material were measured by the potassium dichromate‐sulfuric acid oxidation; N and P concentrations of soil and plant tissue were measured by the diffusion method plus semi‐micro open method and the molybdenum antimony anti‐colorimetric method, respectively; soil AN and AP were measured by the alkali hydrolysis diffusion method and the colorimetry method, respectively (Bao, 2000). Moreover, the R/S ratio was the ratio of root biomass to the sum of stem and leaf biomass; the C, N, and P acquisition were calculated by multiplication of each plant tissue nutrient concentration by each plant tissue biomass and then summing them together.

### 2.3 Mycorrhizal dependency

The mycorrhizal dependency (MD) of the two species in intraspecific competition or interspecific competition was determined according to Van Der Heijden (2002) as follows:

$$\mathrm{MD}=1-\left(b/\bar{a}\right),$$

where $\bar{a}$ is the average biomass of the M^+^ treatment, and *b* is the biomass of the M^−^ treatment.

### 2.4 Competitive effect size

In order to assess the effect size of the competition, the response ratio (LnRR) was calculated by log‐transforming trait values of height, diameter, biomass, C, N, and P acquisition for invasive *E. adenophorum* and native *E. lindleyanum* according to Hedges et al. (1999) as follows:

$$\text{LnRR}=\ln\left(B_{0}/B_{W}\right),$$

where *B*_0_ is the mean trait values including height, diameter, biomass, C, N, and P acquisition under the intraspecific competition of the two species across the six replicates, and *B*_W_ is the trait values under the interspecific competition of the two species within each replication. The value of LnRR is symmetrical around zero. The positive value indicates inhibition, the negative value indicates facilitation, and zero indicates neutral.

### 2.5 Competitive intensity and competitiveness index

Relative yield (RY) was used to assess the relative competitive intensity between intra‐ and interspecific competition of invasive *E. adenophorum* and native *E. lindleyanum* (Keddy et al., 1994). The competitive balance index (CB) was used to measure the competitive ability of the two species (Wilson, 1988). The calculation formulas were as follows:

$${\mathrm{RY}}_{\mathrm{Ea}}=\frac{Y_{\mathrm{ab}}}{Y_{a}};{\mathrm{RY}}_{\mathrm{El}}=\frac{Y_{\mathrm{ba}}}{Y_{b}},$$

$$\mathrm{CB}=\ln\frac{{\mathrm{RY}}_{\mathrm{Ea}}}{{\mathrm{RY}}_{\mathrm{El}}},$$

where *Y*_ab_ represents the single‐plant trait values including height, diameter, biomass, C, N, and P acquisition of the invasive *E. adenophorum* (a) when in competition with the native *E. lindleyanum* (b), *Y*_a_ represents the mean trait values of the single plant for invasive *E. adenophorum* under intraspecific competition; *Y*_ba_ represents the single‐plant trait values of the native *E. lindleyanum* (b) when in competition with the invasive *E. adenophorum* (a), *Y*_b_ represents the mean trait values of single plant for native *E. lindleyanum* under intraspecific competition. Specifically, RY < 1 suggests that the intensity of interspecific competition is stronger than the intraspecific competition, RY > 1 suggests that the intensity of interspecific competition is lower than the intraspecific competition, and RY = 1 suggests that the intensity of intraspecific and interspecific competition is equal. Additionally, CB > 0 suggests that the invasive *E. adenophorum* has a higher competitiveness than native *E. lindleyanum*, CB < 0 suggests that the native *E. lindleyanum* has a higher competitiveness than invasive *E. adenophorum*, and CB = 0 suggests that the two species are equally competitive.

### 2.6 Statistical analysis

All statistical analyses were performed using SPSS 26.0 (SPSS Inc.) software. All raw data were tested for normality and homogeneity tests of variance before analyses to ensure the reliability of subsequent analyses. We used two‐way ANOVA to test the effects of AM fungus (M^+^ and M^−^) and competition (Intra‐ and Inter‐) treatments and their interactions (M × C) on the diameter, height, biomass, R/S ratio, C, N, and P acquisition. Significant differences between M^+^ and M^−^, Intra‐ and Inter‐ on soil pH, SOC, TN, AN, TP, AP, root mycorrhizal colonization, MD, diameter, height, biomass, R/S ratio, C, N, P acquisition, LnRR, RY, and CB were determined using Tukey's test for multiple comparison test at the .05 level. Further, we used principal component analysis (PCA) and correlation matrix to explore the relationship between soil physicochemical properties and plant traits. All graphs were created using Origin 2023 (OriginLab Co.) software.

## 3 RESULTS

### 3.1 Root mycorrhizal colonization and mycorrhizal dependency of both plants

Both invasive *E. adenophorum* and native *E. lindleyanum* showed different levels of root mycorrhizal colonization after inoculation with the AM fungus, whereas roots that had not received the inoculation were not seen to be colonized (Figure S1a–d). Invasive *E. adenophorum* and native *E. lindleyanum* had a high root mycorrhizal colonization (between 54.37% and 70.30%) after being inoculated with AM fungus (Table 1). In contrast to native *E. lindleyanum*, the root mycorrhizal colonization of invasive *E. adenophorum* under Intra‐ treatment was greater than under Inter‐ treatment (Table 1). The MD of invasive *E. adenophorum* and native *E. lindleyanum* in Intra‐ competition was significantly greater than in Inter‐ competition (Table 1). Native *E. lindleyanum* had a significantly higher root mycorrhizal colonization and MD than invasive *E. adenophorum* under Inter‐ treatment, indicating that the native *E. lindleyanum* had more positive response to AM fungi than invasive *E. adenophorum* when competing.

**TABLE 1:** The root mycorrhizal colonization and mycorrhizal dependency of invasive plant *Eupatorium adenophorum* and native plant *Eupatorium lindleyanum*.

Species	Treatments	Root mycorrhizal colonization (%)	Mycorrhizal dependency (MD; %)
*E. adenophorum*	Intra‐	60.85 ± 2.52 aα	85.50 ± 0.004 aα
	Inter‐	54.37 ± 1.93 aβ	47.67 ± 0.03 bβ
*E. lindleyanum*	Intra‐	66.27 ± 1.69 aα	72.83 ± 0.02 aβ
	Inter‐	70.30 ± 2.52 aα	62.17 ± 0.02 bα

*Note*: The different letters (a, b) indicate significant differences between Intra‐ and Inter‐ treatments of invasive *E. adenophorum* and native *E. lindleyanum* (*p* < .05); different Greek letters (α, β) indicate significant differences between invasive *E. adenophorum* and native *E. lindleyanum* (*p* < .05).

### 3.2 Growth and R/S ratio of both plants under AM fungus and competition

**FIGURE 2:**
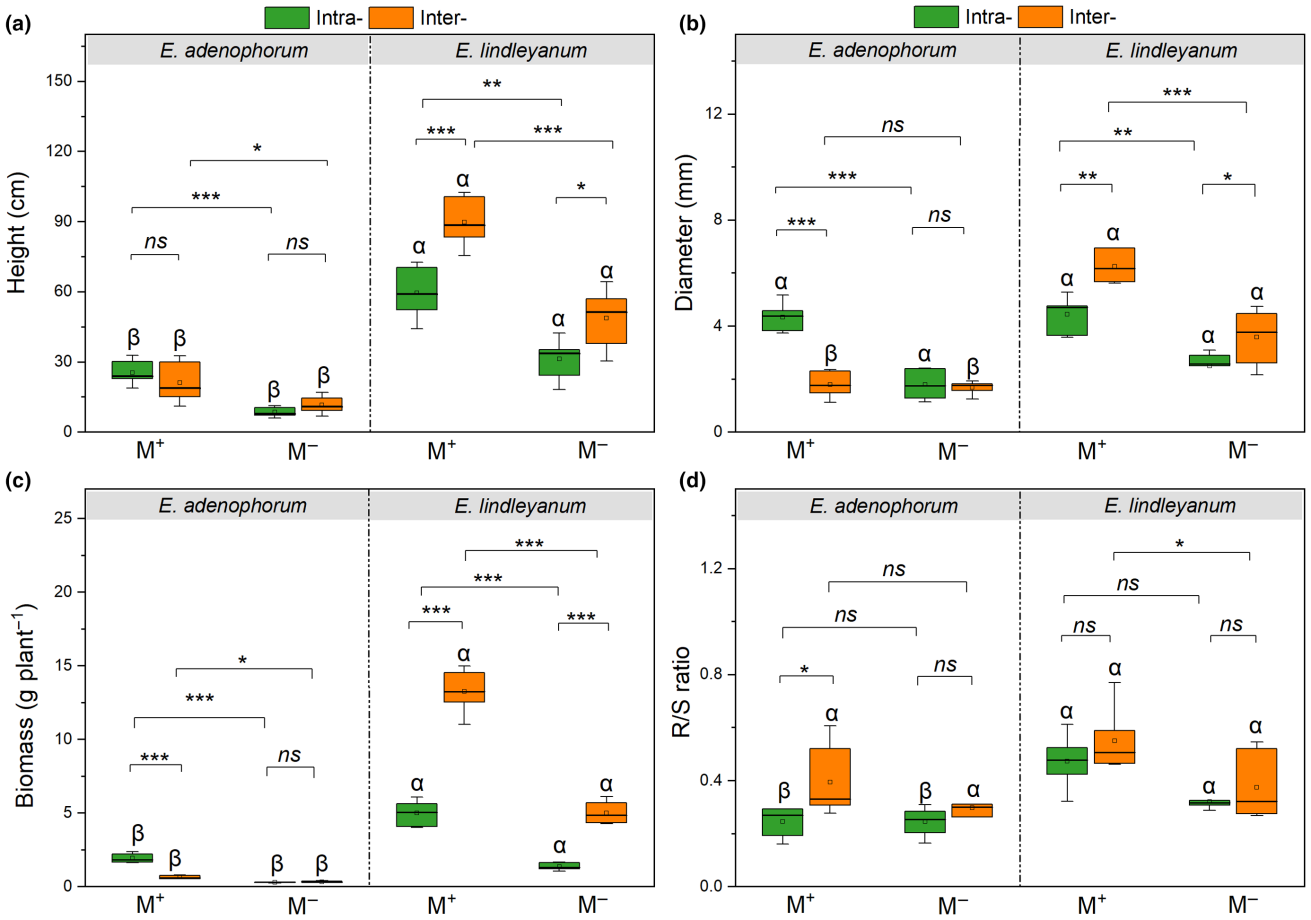
The height, diameter, biomass, and R/S ratio of invasive plant *Eupatorium adenophorum* and native plant *Eupatorium lindleyanum*. Intra‐, intraspecific competition; Inter‐, interspecific competition; M^+^, with AM fungus; M^−^, without AM fungus. The * indicates a significant difference (*p* < .05), the ** and *** (*p* < .01 and *p* < .001) indicate an extremely significant difference, and the *ns* (*p* > .05) indicates a non‐significant difference between M^+^ and M^−^ or Intra‐ and Inter‐; the different Greek letters (α, β) indicate significant differences between invasive *E. adenophorum* and native *E. lindleyanum* (*p* < .05) under M^+^ and M^−^ treatments or Intra‐ and Inter‐ conditions. The error bars represent standard error.

The AM fungus, competition treatments, and their interaction of M × C differently impacted the height, diameter, biomass, and R/S ratio of invasive *E. adenophorum* and native *E. lindleyanum* (Table 2). AM fungus significantly improved the height, diameter, and biomass of the two species in Intra‐ and Inter‐ conditions (Figure 2a–c), and significantly increased the R/S ratio of native *E. lindleyanum* in Inter‐ condition (Figure 2d). For invasive *E. adenophorum* with M^+^, the height, diameter, and biomass in Intra‐ treatment were greater than in Inter‐ treatment (Figure 2a–c). For native *E. lindleyanum* with M^+^ and M^−^ treatments, the height, diameter, and biomass in Inter‐ treatment were significantly greater than in Intra‐ treatment (Figure 2a–c). The R/S ratio of the two species under Inter‐ was higher than under Intra‐ for M^+^ and M^−^ treatments (Figure 2d). In interspecific competition, the height, diameter, and biomass of native *E. lindleyanum* were significantly higher than invasive *E. adenophorum* (Figure 2a–c), indicating that the native *E. lindleyanum* had greater growth than invasive *E. adenophorum* when competing.

**TABLE 2:** The two‐way ANOVA for the effects of AM fungus (M^+^ vs. M^−^) and competition (Intra‐ vs. Inter‐) treatments on the height, diameter, biomass, and R/S ratio of invasive plant *Eupatorium adenophorum* and native plant *Eupatorium lindleyanum*.

Species	Treatments	df	Height		Diameter		Biomass		R/S ratio	
			*F*	*p*	*F*	*p*	*F*	*p*	*F*	*p*
*E. adenophorum*	M	1	35.679	<.001	47.652	<.001	210.432	<.001	1.825	.192
	C	1	0.079	.782	47.059	<.001	87.360	<.001	7.459	<.05
	M × C	1	2.816	.109	39.687	<.001	101.065	<.001	1.713	.205
*E. lindleyanum*	M	1	62.757	<.001	55.092	<.001	247.751	<.001	16.074	<.001
	C	1	29.641	<.001	21.785	<.001	249.813	<.001	2.603	.122
	M × C	1	2.124	.160	1.280	.271	37.383	<.001	0.072	.792

*Note*: M × C represents the interaction between M and C. In addition, the “<.05” indicates a significant effect, and the “<.01” and “<.001” indicate an extremely significant effect.

Abbreviations: AM, arbuscular mycorrhizal; C, competition treatment; M, AM fungus treatment.

### 3.3 C, N, and P acquisition of both plants under AM fungus and competition

**FIGURE 3:**
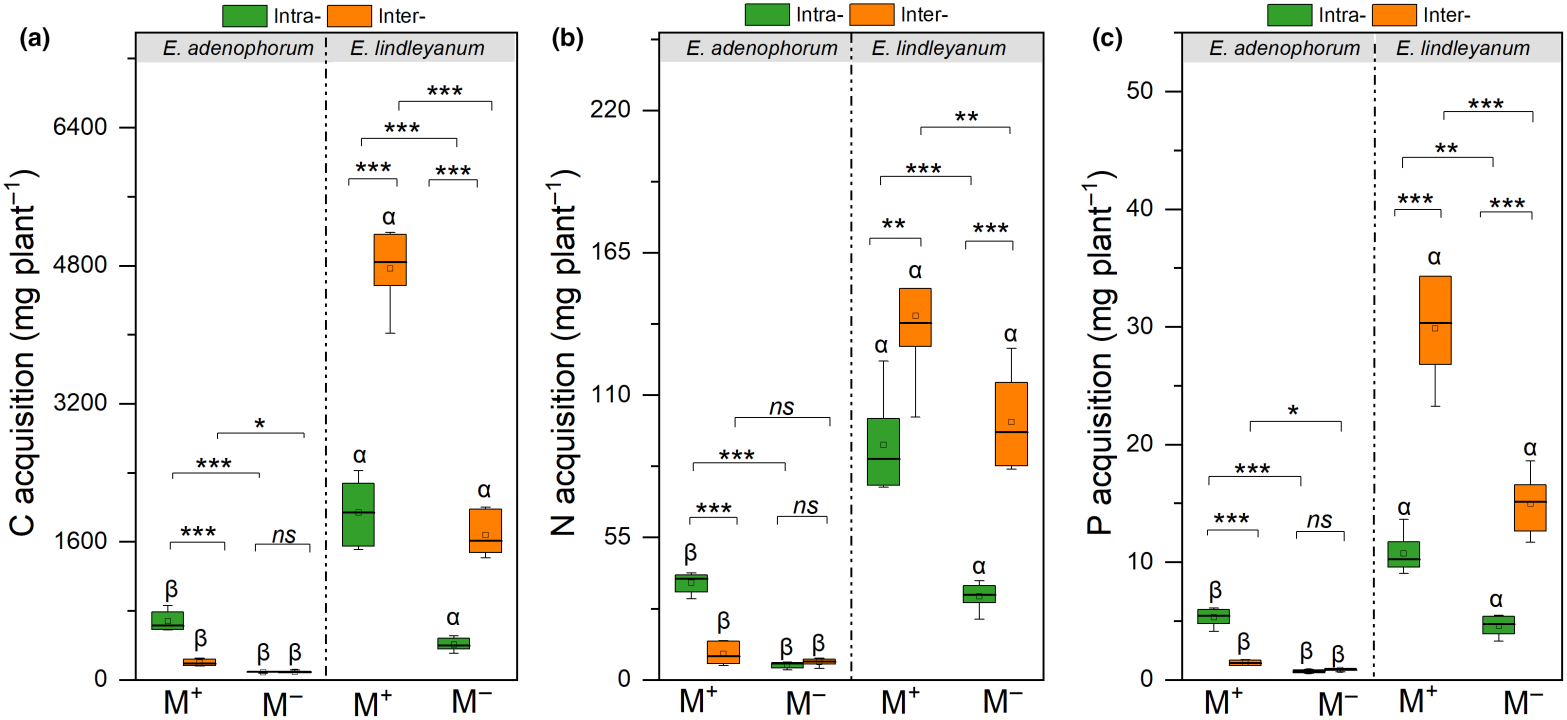
The C, N, and P acquisition of invasive plant *Eupatorium adenophorum* and native plant *Eupatorium lindleyanum*. M^+^ and M^−^; Intra‐ and Inter‐, implications are the same as in Figure 2; the *, **, *** and *ns*, and the Greek letters α and β above the bars, implications are the same as in Figure 2.

The AM fungus and competition treatments significantly influenced the C, N, and P acquisition of invasive *E. adenophorum* and native *E. lindleyanum*, the interaction of M × C significantly influenced the C, N, and P acquisition of invasive *E. adenophorum* and the C and P acquisition of native *E. lindleyanum* (Table 3). The AM fungus significantly increased the C, N, and P acquisition of the two species (Figure 3a–c). For invasive *E. adenophorum* with M^+^, the C, N, and P acquisition under Intra‐ treatment were significantly higher than under Inter‐ treatment (Figure 3a–c). For native *E. lindleyanum* with M^+^ and M^−^ conditions, the C, N, and P acquisition in Inter‐ competition were significantly higher than in Intra‐ competition (Figure 3a–c). Comparing the two species, the C, N, and P acquisition of native *E. lindleyanum* were significantly higher than invasive *E. adenophorum* (Figure 3a–c), indicating that the native *E. lindleyanum* had greater nutrient acquisition than invasive *E. adenophorum* when competing.

**TABLE 3:** The two‐way ANOVA for the effects of AM fungus (M^+^ vs. M^−^) and competition (Intra‐ vs. Inter‐) treatments on the C, N, and P acquisition of invasive plant *Eupatorium adenophorum* and native plant *Eupatorium lindleyanum*.

Species	Treatments	df	C acquisition		N acquisition		P acquisition	
			*F*	*p*	*F*	*p*	*F*	*p*
*E. adenophorum*	M	1	185.860	<.001	191.717	<.001	237.335	<.001
	C	1	86.273	<.001	108.716	<.001	122.824	<.001
	M × C	1	89.337	<.001	127.592	<.001	140.508	<.001
*E. lindleyanum*	M	1	299.425	<.001	38.895	<.001	88.441	<.001
	C	1	235.828	<.001	54.317	<.001	173.521	<.001
	M × C	1	33.932	<.001	1.213	.284	15.267	<.01

*Note*: M × C represents the interaction between M and C. In addition, the “<.05” indicates a significant effect, and the “<.01” and “<.001” indicate an extremely significant effect.

Abbreviations: AM, arbuscular mycorrhizal; C, competition treatment; M, AM fungus treatment.

### 3.4 The competitive intensity and competitive ability of both plants

**FIGURE 4:**
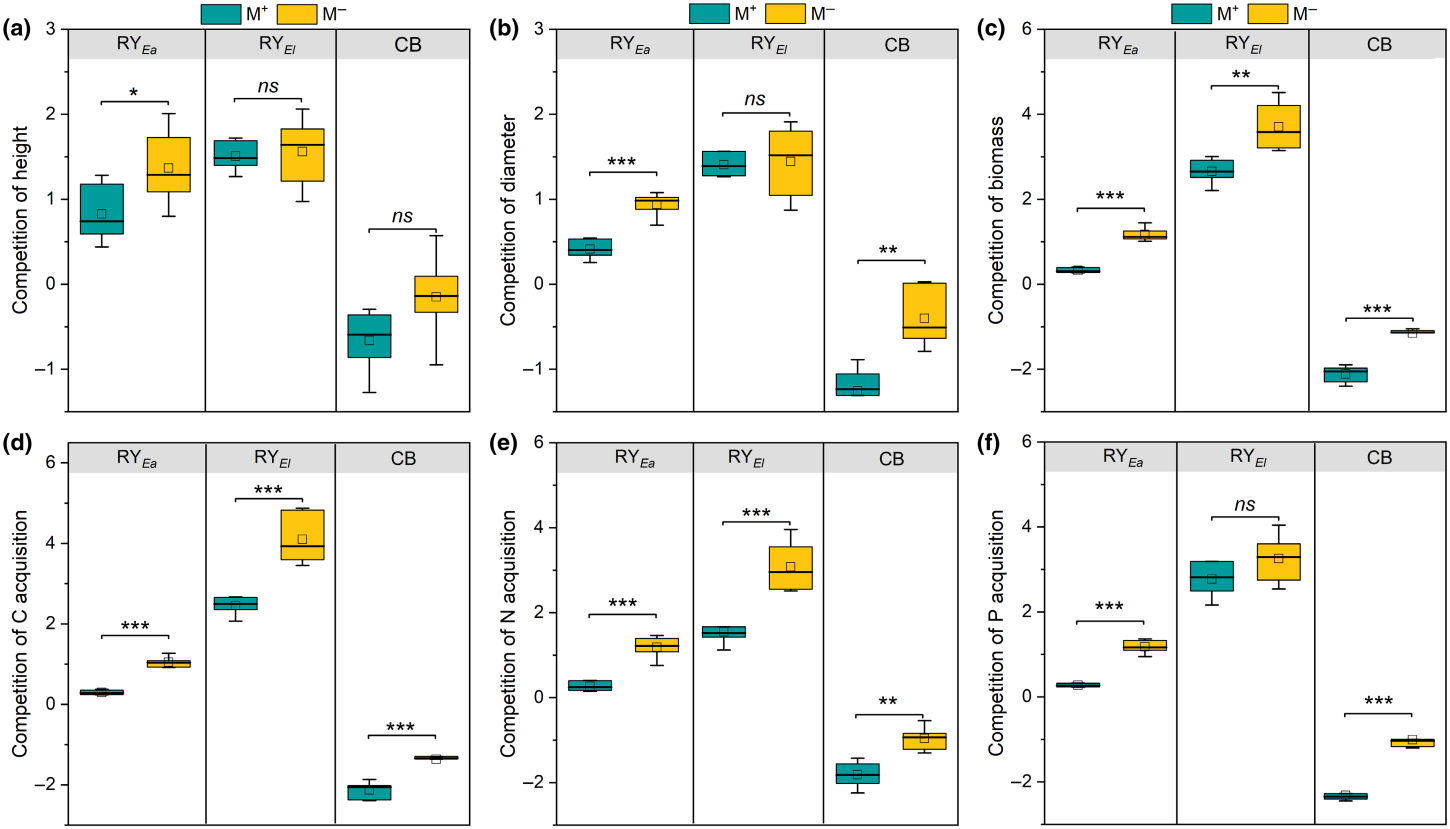
The competition intensity and competitive ability of invasive plant *Eupatorium adenophorum* and native plant *Eupatorium lindleyanum*. M^+^ and M^−^ implications are the same as in Figure 2; CB, competitive balance index; RY, relative yield. The *, **, ***, and *ns* implications are the same as in Figure 2.

The RY of height, diameter, biomass, C, N, and P acquisition for the two species under M^−^ treatment was higher than under M^+^ treatment, and the CB of diameter, biomass, C, N, and P acquisition was significantly different between M^+^ and M^−^ treatments (Figure 4a–f). For invasive *E. adenophorum*, the RY > 1 under M^−^ treatment and RY < 1 under M^+^ treatment, suggesting that the intensity of Intra‐ competition was stronger than Inter‐ competition under M^−^ treatment, and the intensity of Inter‐ competition was greater than Intra‐ competition under M^+^ treatment (Figure 4a,c,d–f). For native *E. lindleyanum*, the RY > 1 under M^+^ and M^−^ treatments, suggesting that the intensity of Intra‐ competition was greater than the Inter‐ competition (Figure 4a–f). In addition, the CB < 0 under M^+^ and M^−^ treatments, meaning that the competitiveness in growth and nutrition of native *E. lindleyanum* was higher than invasive *E. adenophorum* (Figure 4a–f). Meanwhile, the AM fungus significantly promoted the competitiveness of native *E. lindleyanum* relative to invasive *E. adenophorum* (Figure 4a–f). Additionally, the LnRR of height, diameter, biomass, C, N, and P acquisition of invasive *E. adenophorum* under M^+^ treatment was significantly higher than under M^−^ treatment (Figure S2). Under M^+^ treatment, the Inter‐ competition inhibited the growth and nutrition of invasive *E. adenophorum* and promoted the growth and nutrition of native *E. lindleyanum* relative to Intra‐ competition; under M^−^ treatment, the Inter‐ competition promoted the growth of native *E. lindleyanum* relative to Intra‐ competition (Figure S2).

### 3.5 Correlation analysis between soil physicochemical properties and plant traits

**FIGURE 5:**
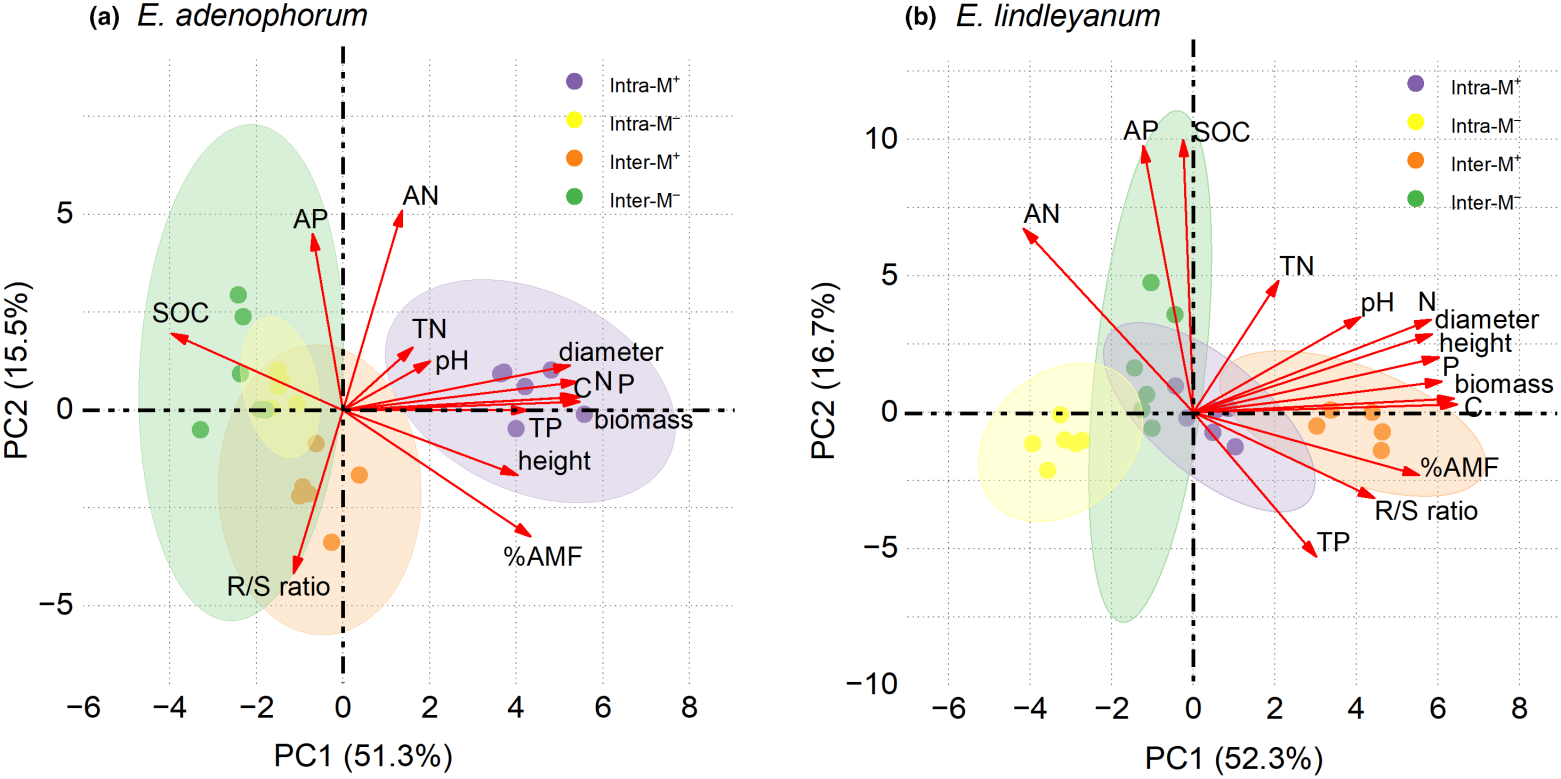
The principal component analysis (PCA) for soil physicochemical properties and plant traits. The PCA involves the relationships between the soil physicochemical properties (soil pH, SOC, TN, AN, TP, AP) and plant traits (root mycorrhizal colonization: %AMF, height, diameter, biomass, C, N and P acquisition) of invasive *Eupatorium adenophorum* and native *Eupatorium lindleyanum*.

**FIGURE 6:**
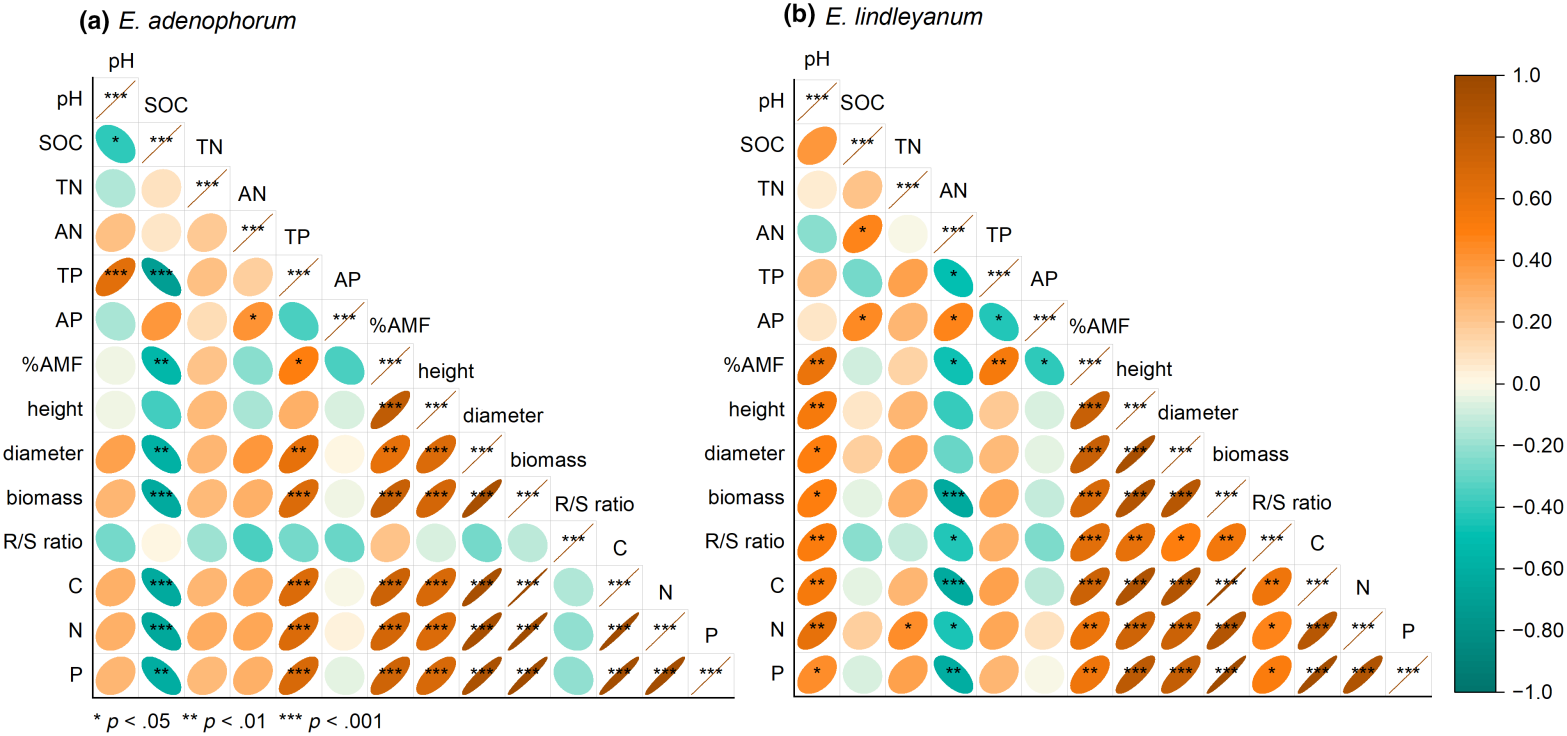
The correlation between plant traits and soil physicochemical properties. AN, alkaline nitrogen; AP, alkaline phosphorus; SOC, soil organic carbon; TN, total nitrogen; TP, total phosphorus.

The competition style and AM fungus had a differential impact on the soil physicochemical parameters involving the soil pH, SOC, TN, AN, TP, and AP (Table S1). Principal component analysis (PCA) revealed the relationship between soil physicochemical parameters and plant traits (Figure 5). For invasive *E. adenophorum*, the first and second principal components (PC1 and PC2) explained 51.3% and 15.5% of the variance, respectively (Figure 5a). The first PCA and correlation matrix showed that the height, diameter, biomass, C, N, and P acquisition of invasive *E. adenophorum* were significantly positively related to the soil TP and %AMF, and significantly negatively related to SOC (Figures 5a and 6a). For native *E. lindleyanum*, the first and second principal components (PC1 and PC2) explained 52.3% and 16.7% of the variance, respectively (Figure 5b). The second PCA and correlation matrix showed that the height, diameter, biomass, R/S ratio, C, N, and P acquisition of native *E. lindleyanum* were significantly positively related to the soil pH and %AMF and significantly negatively related to soil AN (Figures 5b and 6b).

## 4 DISCUSSION

### 4.1 Differences in competition for growth and nutrition between native and invasive plants

Our research demonstrates that AM fungus significantly enhanced the growth and nutrition of both invasive *E. adenophorum* and native *E. lindleyanum* under different competition conditions (Figures 2a–c and 3a–c). These findings are consistent with previous studies that have shown that AM fungi colonizing the plant roots can promote host plant growth and improve nutrition acquisition by widening the space for the plant root system to capture soil resources (He, 2019; He et al., 2019; Li et al., 2022). Many studies have shown that invasive plants possess a greater competitive advantage compared to native plants (Dong et al., 2021; Shen et al., 2020; Zhang et al., 2018). However, in this study, the native *E. lindleyanum* had a higher competitiveness than the congeneric invasive *E. adenophorum* regardless of whether they were inoculated with AM fungus or not (Figure 4a–f), which was partially inconsistent with H1 that invasive plants exhibit higher competitiveness than native plants. This paradoxical result may be attributed to the heterogeneity of paired plant partners (Lanfranco et al., 2018).

Most previous conclusions came from research on pairs of heterogeneous native and invasive plants with different life histories, sizes, and taxonomies (MacDougall et al., 2009; Vila & Weiner, 2004) and even with different families (Dong et al., 2021; Zhang et al., 2018). This diversity among paired plant partners could potentially contribute to the inconsistency in findings and add complexity to understanding the competitive dynamics between invasive and native species. In this study, there were no significant differences in root mycorrhizal colonization between the congeneric invasive *E. adenophorum* and the native *E. lindleyanum* under intraspecific competition, indicating that both plant species respond similarly to mycorrhizae. This phenomenon can be explained by the high similarity between the two plant species, as native species *E. lindleyanum* is phylogenetically related to the invasive *E. adenophorum* (Li et al., 2017; Sun et al., 2022). Therefore, the native *E. lindleyanum* was probably more competitive than the congeneric invasive *E. adenophorum* as it was highly similar to the invasive *E. adenophorum*. A field experiment supported this finding, which suggested that native plants with similar functions to invasive plants are more resistant to invasion (Young et al., 2009). A controlled experiment also showed that native plant communities with a closer phylogenetic relationship to the plant invader exhibited higher resistance to the invasion (Wang et al., 2022). Therefore, our findings potentially provide support for Darwin's naturalization and limiting similarity hypotheses, indicating that native plants closely related to congenic invasive plants may effectively control plant invasions (Darwin, 1859; MacArthur & Levins, 1967). Meanwhile, some other studies also suggest that convergent evolution may lead to ecological niche overlap between distantly related invasive and native plants, resulting in intense competition that can impact the competition outcomes (Cleland et al., 2011; Wang et al., 2021). However, further investigation through subsequent experiments is required to explore this phenomenon in more depth.

Furthermore, as shown in Figure 2d, the native *E. lindleyanum* exhibited a higher R/S ratio than the invasive *E. adenophorum* under different competition conditions, regardless of inoculation with or without AM fungus. It indicates that the native *E. lindleyanum* tends to invest more proportion of photosynthetic products toward the belowground for root building, thus obtaining more nutrients compared to invasive *E. adenophorum*. This allocation may enable the native species to grow at a faster rate than the invasive species (Mašková & Herben, 2018), which leads to the larger size advantage exhibited by native *E. lindleyanum* over invasive *E. adenophorum*, evidenced by their greater height, diameter, and biomass (Figure 2a–c). Research showed that plants with larger sizes tend to be more competitive in terms of accessing resources such as sunlight, water, and nutrients (Tracey & Aarssen, 2011). Consequently, the growth and size advantage of native *E. lindleyanum* may also be one of the factors bolstering their competitiveness compared to invasive *E. adenophorum*.

### 4.2 Differences in the competitive advantage between invasive and native plants in association with AM fungi

The native *E. lindleyanum* was more competitive than invasive *E. adenophorum* despite AM fungus; however, the AM fungus significantly improved this competitiveness (Figure 4a–f). It was partially inconsistent with H1 that AM fungi promote this competitive advantage of the invasive species compared to native species. Differences in the mycorrhizal association between plant species could potentially explain this phenomenon. Research shows that the competitive advantage of native plants against invasive plants is largely dependent on the mycorrhizal association (Huangfu et al., 2019; Mchaffie & Maherali, 2020). In this study, the root mycorrhizal colonization and mycorrhizal dependency of native *E. lindleyanum* were higher than those of invasive *E. adenophorum* under interspecific competition (Table 1). This indicates that plants with a higher mycorrhizal dependence receive noticeably more benefits from AM fungi than plants with a lower mycorrhizal dependence when species compete (Cheng, Zhang, et al., 2022; Greipsson & DiTommaso, 2006; Majewska et al., 2017). A review holds these findings, which claim that there is a positive correlation between plant mycorrhizal dependence and the amount of P obtained from AM fungi, and plant‐to‐plant C transport through the mycorrhizal network is preferentially directed toward plants with the highest mycorrhizal dependence (Van Der Heijden, 2002). Intriguingly, the invasive *E. adenophorum* was more mycorrhizal dependent than the native *E. lindleyanum* under intraspecific competition (Table 1), which suggested the plant invaders have a more positive response to AM fungi than natives when they grow alone (Aslani et al., 2019; Guo et al., 2022).

Plant invasion can impact soil physicochemical properties, such as soil pH, water, and nutrient pools, which can in turn affect the growth and competitive dynamics of both invasive and native plants (Stefanowicz et al., 2018; Zubek et al., 2022). A previous study showed that soil physicochemical parameters, including soil pH and SOC, are the major factors contributing to the successful invasion of alien plants (Wamelink et al., 2018). In this study, the growth and nutrition of invasive *E. adenophorum* were notably negatively related to the SOC and notably positively related to the soil TP but not notably related to soil pH; in contrast, the growth and nutrition of native *E. lindleyanum* were notably positively associated with the soil pH and notably negatively related to the soil AN but not notably related to SOC (Figures 5 and 6). Therefore, the competitive advantage difference between invasive and native plants may be primarily influenced by their effects on soil pH and SOC, potentially associated with root mycorrhizal colonization. This finding aligns with the observations of Soti et al. (2015), who observed that soil pH significantly influenced the root mycorrhizal colonization of the invasive *Lygodium microphyllum*. Our result exhibits a notable positive association between soil pH and root mycorrhizal colonization in native *E. lindleyanum*, while no notable relationship was observed in invasive *E. adenophorum*. Additionally, there was a notable positive correlation between root mycorrhizal colonization and the growth and nutrition of both species (Figures 5 and 6). Therefore, soil physicochemical properties, including soil pH and SOC, may indirectly influence plant growth and the competition between invasive and native plants by affecting mycorrhizal colonization (Laurindo et al., 2021).

### 4.3 Differential competition intensities of intraspecific and interspecific in association with AM fungi

Intra‐ and interspecific competition intensity exert a significant influence on plant growth and nutrient acquisition (Mangla et al., 2011). Generally, intense competition constrains the capacity of plants to access resources (Yang et al., 2019). Our result supported this point, which showed that the intensity of intraspecific competition was higher than the interspecific competition for both invasive *E. adenophorum* and native *E. lindleyanum* under M^−^ treatment (Figure 4). This finding was inconsistent with our H2 and indicates that both plant species experienced greater growth and nutrition during interspecific competition than during intraspecific competition under M^−^ treatment (Figures 2a–c and 3a–c). Previous research has suggested that AM fungi can regulate the intensity of both intra‐ and interspecific competitions (Guo et al., 2021). Contrary to the native *E. lindleyanum*, the intensity of interspecific competition was higher than intraspecific competition for invasive *E. adenophorum* under the M^+^ treatment (Figure 4a–f). This finding is contradictory to classical competition theory, which states that the intensity of intraspecific competition ought to be greater than the interspecific competition due to conspecific individuals having more similar resource requirements than heterospecific individuals (Hart & Marshall, 2009). In particular, contrary to classical competition theory, interspecific competition intensity between native and non‐native plants may outweigh intraspecific competition intensity (Sheppard & Burns, 2014). This phenomenon may be attributed to AM fungi stimulating the preemption of scarce resources by both invasive and native plants (Duell et al., 2021), resulting in stronger interspecific competition between native and invasive species occupying similar ecological niches or habitats (Shen et al., 2022; Sheppard & Burns, 2014). Consequently, the intense interspecific competition negatively affected the growth of invasive *E. adenophorum* compared to intraspecific competition (Figure S2).

Notably, the AM fungus reduced both intra‐ and interspecific competition intensities of invasive *E. adenophorum* and native *E. lindleyanum* (Figure 4a–f). Research has shown that AM fungi can establish connections among different individuals within a plant community (Tedersoo et al., 2020), thereby regulating the nutrient transport between invasive and native plants in the soil and subsequently affecting plant competition (Shen et al., 2020). Therefore, AM fungi may enhance resource allocation and utilization in both species by alleviating the intensity of both intra‐ and interspecific competitions, although further investigation using isotope tracing is warranted. Intriguingly, we observed that invasive *E. adenophorum* displayed the highest mycorrhizal dependency under intraspecific competition, and the lowest mycorrhizal dependency under interspecific competition compared to other treatments (Table 1). This finding may potentially explain the invasive species' propensity to form monodominant communities (Vila & Weiner, 2004), as *E. adenophorum* derives greater benefits from intraspecific competition, particularly in terms of mycorrhizal benefits, which enhances their expansion. However, as a pot experiment, there may be some potential limitations in this study. Pot experiments, as controlled indoor experiments, may not be able to fully replicate the complexities and interactions found in natural environments. Therefore, it is crucial to recognize that extrapolating these findings to natural ecosystems should be done with caution. Thus, in the future, we will conduct further research under outdoor field conditions to validate and expand upon our findings.

## 5 CONCLUSIONS

In this study, the AM fungus significantly improved the growth and nutrient acquisition of both the invasive plant *E. adenophorum* and the native plant *E. lindleyanum*. Compared to intraspecific competition, interspecific competition with native *E. lindleyanum* inhibited the growth and nutrition of invasive *E. adenophorum* when inoculated with AM fungus. Meanwhile, native *E. lindleyanum* had significantly higher competitiveness than invasive *E. adenophorum* in growth and nutrition, and AM fungus significantly increased the competitiveness of native *E. lindleyanum* relative to invasive *E. adenophorum*. Overall, AM fungus enhances the competitive advantage of native *E. lindleyanum* compared to invasive *E. adenophorum* in growth and nutrition, which emphasizes the importance of AM fungi in helping native plants resist the invasion of alien plants, further contributing to understanding the plant invasion prevention mechanism.

## AUTHOR CONTRIBUTIONS

**Kaiping Shen:** Conceptualization (equal); software (equal); writing – original draft (equal). **Yuejun He:** Funding acquisition (equal); writing – review and editing (equal). **Tingting Xia:** Resources (equal). **Yun Guo:** Data curation (equal). **Bangli Wu:** Data curation (equal). **Xu Han:** Data curation (equal). **Hongchun Chen:** Formal analysis (equal); methodology (equal). **Yan Zhao:** Formal analysis (equal); methodology (equal). **Pan Wu:** Supervision (equal). **Yuan Liu:** Supervision (equal).

## CONFLICT OF INTEREST STATEMENT

The authors declare no conflict of interest.

## Supporting information


Appendix S1

## Data Availability

The dataset used in this study is available at the following: https://doi.org/10.6084/m9.figshare.23257181.
