# Supplementary material for: Arbuscular mycorrhizal fungi improve the competitive advantage of a native plant relative to a congeneric invasive plant in growth and nutrition
Source: Ecol Evol. 2024 May 21;14(5):e11459. doi: 10.1002/ece3.11459 (PMC11106688; doi:10.1002/ece3.11459)

**TABLE S1 The soil physicochemical properties.** Abbreviations: SOC = soil organic carbon, TN = total nitrogen, AN = alkaline nitrogen, TP = total phosphorus, AP = alkaline phosphorus. Intra- = Intraspecific competition; Inter- = Interspecific competition; *Ea* = *E. adenophorum*; *El* = *E. lindleyanum*; M^+^ = with AM fungus; M^–^ = without AM fungus. The different letters (X, Y) indicate significant differences between M^+^ and M^-^ treatments (*P* < 0.05); the different letters (a-c) indicate significant differences among Intra-*Ea*, Intra-*El* and Inter- treatments under M^+^ treatment (*P* < 0.05); the different letters (α-γ) indicate significant differences among Intra-*Ea*, Intra-*El* and Inter treatments under M^-^ treatment (*P* < 0.05).

| Treatments | | pH | SOC (g·kg^-1^) | TN (g·kg^-1^) | AN (mg·kg^-1^) | TP (g·kg^-1^) | AP (mg·kg^-1^) |
| --- | --- | --- | --- | --- | --- | --- | --- |
| Intra-*Ea* | M^+^ | 7.59 ± 0.01Xa | 39.11 ± 2.15Yc | 0.49 ± 0.04Xa | 342.69 ± 3.16Xa | 0.94 ± 0.01Xa | 13.43 ± 0.97Xa |
|  | M^-^ | 7.62 ± 0.03Xα | 53.53 ± 3.05Xβ | 0.43 ± 0.02Xα | 332.26 ± 10.16Xα | 0.81 ± 0.03Yα | 11.68 ± 0.56Xβ |
| Intra-*El* | M^+^ | 7.62 ± 0.10Xa | 64.22 ± 2.81Xa | 0.43 ± 003Xa | 321.57 ± 9.42Xa | 0.74 ± 0.04Xb | 10.30 ± 1.02Xa |
|  | M^-^ | 7.06 ± 0.02Yβ | 53.28 ± 1.80Xβ | 0.41 ± 0.02Xα | 326.15 ± 5.32Xα | 0.64 ± 0.04Yβ | 10.07 ± 0.90Xβ |
| Inter- | M^+^ | 7.50 ± 0.01Xa | 54.27 ± 2.57Yab | 0.47 ± 0.02Xa | 292.44 ± 7.10Yb | 0.73 ± 0.04Xb | 10.46 ± 1.39Ya |
|  | M^-^ | 7.49 ± 0.01Xα | 68.92 ± 6.87Xα | 0.45 ± 0.04Xα | 329.72 ± 7.99Xα | 0.59 ± 0.04Yβ | 17.02 ± 1.48Xα |

**FIGURE S1 The microscopic images of mycorrhizal formation.** The microscopic images of mycorrhizal formation (a-d) were taken after the harvest of the receiver seedlings of invasive *E. adenophorum* and native *E. lindleyanum* at the end of the experiment, including the uncolonized root cells, arbuscule, external mycelium, vesicle, and hyphae.


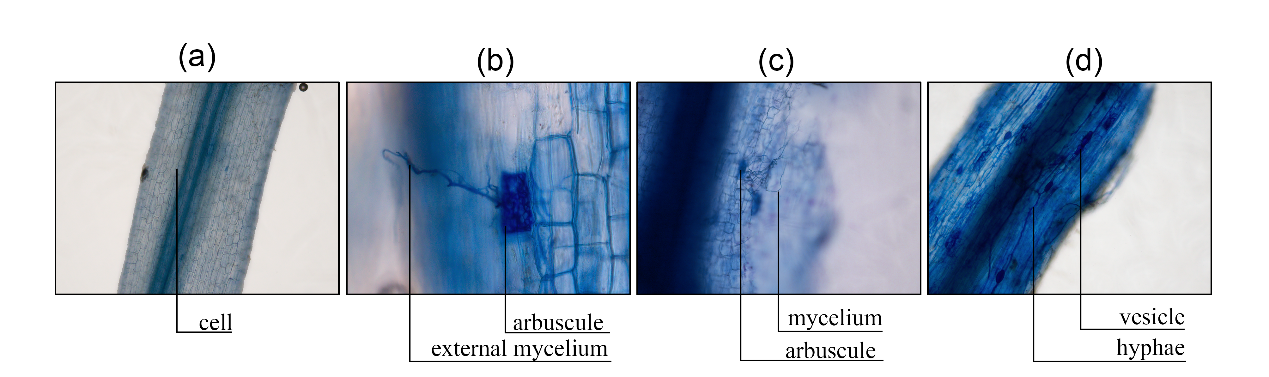


**FIGURE S2 The LnRR for plant growth and nutrition of invasive plant *Eupatorium adenophorum* and native plant *Eupatorium lindleyanum*.** Abbreviations: M^+^ = with AM fungus; M^-^ = without AM fungus. The * indicates a significant difference (*P* < 0.05), the ** and *** (*P* < 0.01 and *P* < 0.001) indicate an extremely significant difference, and the *ns* (*P* > 0.05) indicates a non-significant difference between M^+^ and M^-^ treatments.


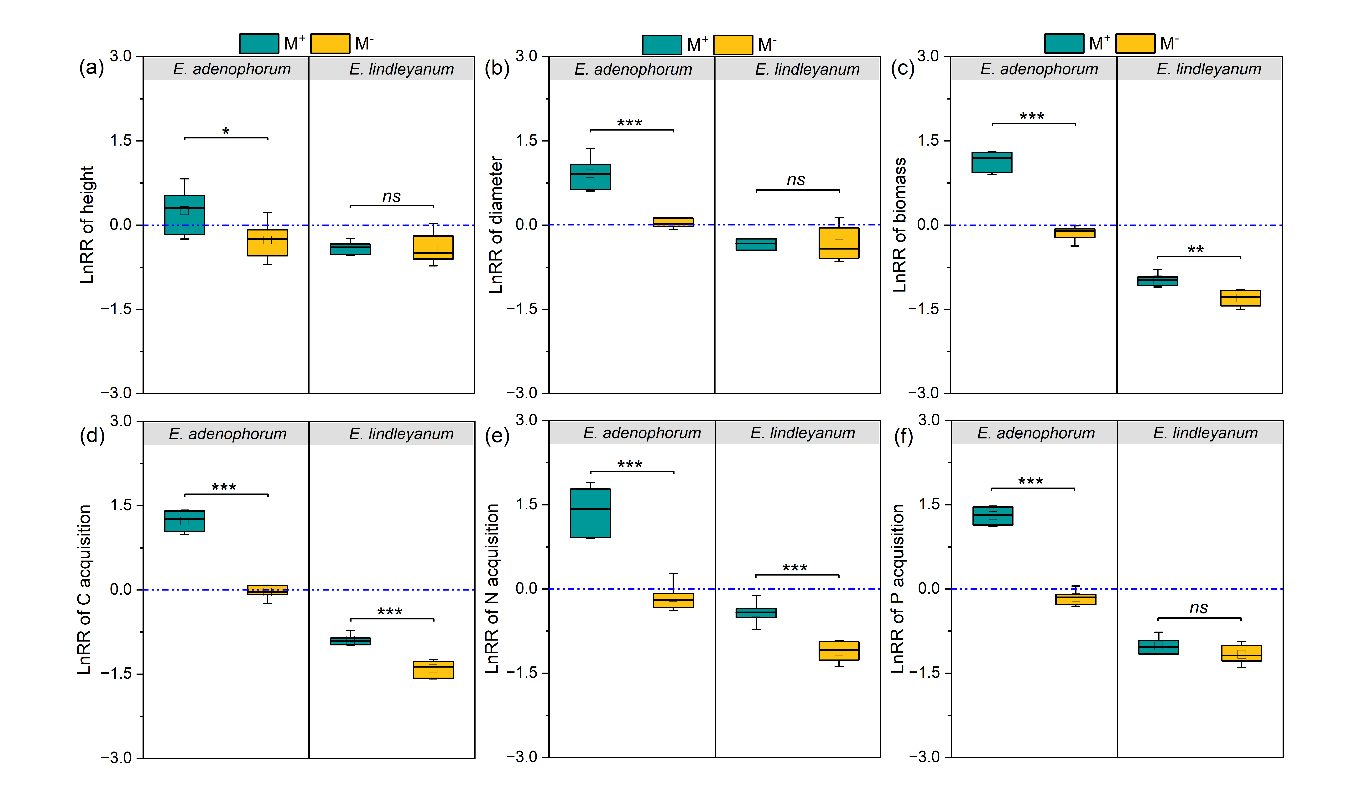

Supplement: Supplementary file 1 — Appendix S1 [file ECE3-14-e11459-s001.docx]
